# Investigate the Influencing Factors of Industrial Design Platform Demand: From the Perspective of Emotional Interaction

**DOI:** 10.3389/fpsyg.2022.892771

**Published:** 2022-05-23

**Authors:** Chenxiao Zhang, Qin Yang, Lei Tong, Rong Zhou

**Affiliations:** ^1^School of Architecture and Artistic Design, University of Science and Technology Liaoning, Anshan, China; ^2^School of Tourism Management, Wuhan Business University, Wuhan, China; ^3^Faculty of Business and Economics, University of Malaya, Kuala Lumpur, Malaysia

**Keywords:** big data, emotional interaction, industrial design service platform, grounded theory, interpretative structural model

## Abstract

With the deep integration of industries brought about by big data technology, users’ design needs are diversifying and individuating. Thanks to big data technology, users’ diverse design needs can be precisely met. Meanwhile, big data can be used to realize emotional interaction for personalized design needs of users, resulting in a better user experience. Using grounded theory to mine user demand text data, this paper investigates the influencing factors of emotional interaction and dynamic resource allocation in the digital design supply chain. The results show that government-driven factors have a direct impact on the demand for industrial design in user emotional interactions. Market factors are the most fundamental in the development of an industrial design service platform, and universities play an important role in this. Furthermore, a lack of market sensitivity stems from a lack of emotional interaction with users, resulting in a schism between industry, university, and research, which has become a major impediment to the development of China’s industrial design industry. This study not only lays the theoretical groundwork for understanding the mechanisms of user emotional interaction on IDSPs, but it also points the way forward for future industrial design service platform development.

## Introduction

Promoting the integration of industry-university research in the design industry has a positive impact on promoting high-quality economic development (Shao X.-F. et al., 2021; Oliva et al., 2022; Zhang H. et al., 2022). Using digital technology to build an industrial design service platform (IDSP) is an effective way to address the current disconnect between Chinese design and the market, even though the Chinese design market is currently experiencing a significant supply and demand gap (Onsman, 2016; FuJun et al., 2018; Xu et al., 2018). Building a digital IDSP can help to dismantle the traditional teaching concept of industrial design as an art discipline and actively explore a development model that integrates industry, academia, and research (Wang L. et al., 2022; Zhang and Ming, 2022). However, current IDSP places a greater emphasis on conceptual design, resulting in ineffective interaction between design solutions and users (da Silva Rodrigues, 2021), making translation of design results difficult (Castelo-Branco et al., 2022; Selicati et al., 2022). Therefore, improving the efficiency of design commercialization necessitates addressing the traditional industrial design process’s lack of effective designer-user interaction (particularly emotional interaction; Jovanovic et al., 2021; Taneri and Dogan, 2021; Zhang X. et al., 2022).

Through industrial design, product innovation promotes industrial upgrading (Shao Q. et al., 2021; Zhu et al., 2021). Building an IDSP in the early, middle, and late stages of product development can provide enterprises and designers with a wealth of user interaction information (Li et al., 2020; Hsiao and Lee, 2021; Christou et al., 2022). Through the IDSP, designers and users interact deeply emotionally, effectively improving the ability to manage the entire product design process (concept innovation, modeling development, and realisability), and thus improving design transformation efficiency (Zivkovic et al., 2015; Corazza et al., 2021; Zhang, 2021). While science and technology provide the foundation for emotional interaction, emotional interaction inspires design creativity, which opens up endless possibilities for science and technology applications (Brand et al., 2015). Consequently, building an IDSP to solve the interaction between designers and users in the collaborative innovation process and promote intelligent matching of design tasks and design resources is an effective way to increase the efficiency of design commercialization (Sørensen, 2008; Kabukcu, 2015; He G. et al., 2020). Through resource integration and demand optimization allocation, distributed heterogeneity and dynamic intelligent matching of design resources can be realized, effectively supporting the manufacturing industry’s transformation and upgrading (Cheng, 2019; An et al., 2020; Nain et al., 2022).

Overall, this paper’s marginal contribution to existing research is primarily in the following areas: First, this paper adds to the current in-depth integration of industrial design and industry-university-research collaborative innovation to promote innovation transformation efficiency; second, it fills a gap in current industrial design research by exploring the internal relationship between industrial design and innovation-driven development from the user’s perspective, and expanding industrial design research beyond pure education and research. Third, the paper examines the current industrial design development trend and offers feasible suggestions for enterprise and university talent training through an in-depth analysis of demand for IDSPs. Fourth, this paper expands on previous research that focused on a single product or series of products, enhancing the practical importance of industrial design for innovation-driven development.

## Literature Review

In the traditional supply chain, the phenomenon of multi-service resource crossing and multi-organization information barriers causes a lag in resource recommendation time, causing enterprises to face the problem of reduced information resource flow efficiency in the industrial interconnection business (Dumitrache et al., 2020; He G. et al., 2020; Jianjia et al., 2021). Product improvement requires innovation in order to gain a competitive advantage in the market (business success; He L. et al., 2020; Wang et al., 2021). Not all small and medium-sized businesses, however, can afford the high cost of innovation (Kori et al., 2021; Zheng et al., 2021). Therefore, the industrial design service platform based on collaborative innovation between industry and university research frequently plays an important role in small and medium-sized business innovation (Lawson, 2005; Shao X.-F. et al., 2021). Design researchers contribute their knowledge and time to the platform to help with product design and manufacturing process innovation (Chowdhury et al., 2021; Liu Z. et al., 2022). Using collaborative design to develop innovative design solutions encourages everyone to share their perspectives and expertise (Mariani and Nambisan, 2021; Benitez et al., 2022). Design thinking encourages the exploration and evaluation of new ideas by opening up new channels of communication (Tiefenbacher, 2019; Paay et al., 2021; Stuedahl et al., 2021). The essence of this phenomenon is that digitalization aids in the intelligent development of industrial design and is a powerful force in promoting the transformation of technological innovation (Sinan Erzurumlu and Erzurumlu, 2015; Bartliff et al., 2020).

### Emotional Interaction and Industrial Design in the Context of Digitalization

Companies are enabling collaborative sharing and personalized services through digital platforms, which has emerged as one of the most important strategies for companies seeking to achieve innovative growth (Matthews et al., 2021; Espinoza Pérez et al., 2022; Liu Z. et al., 2022; Zhang H. et al., 2022). For smart manufacturing, the industrial Internet connects the physical world and cyberspace (Srinidhi et al., 2019; He L. et al., 2020). Furthermore, as a result of user-producer collaboration, production efficiency can be improved, while product design can be more accommodating to users’ emotional needs (Hao et al., 2021; Otto et al., 2021). Therefore, when purchasing products and services, the most important factor to consider is how to provide consumers with an emotional experience that exceeds their expectations through design innovation (Bu et al., 2021; Wehrle et al., 2021).

IDSP enables the virtual clustering of companies and users, allowing dispersed design resources and ideas to overcome geographic space limitations (Ogundoyin and Kamil, 2021; Pivoto et al., 2021), laying the groundwork for emotional interaction between users and companies (Ogundoyin and Kamil, 2021; Pivoto et al., 2021). The traditional industry of manufacturing equipment suppliers has given way to industrial product service system (IPSS) providers as firms’ profitability shifts from products to services (Mourtzis et al., 2021; Yoon et al., 2021). IPSS is a new business model that enables manufacturing as a service by providing products and the services that go with them on a continuous basis (Morgan et al., 2021; Yuan et al., 2021). Users are able to exceed their expectations as a result of this manufacturing as a service, which is enabled by network collaboration with users (Lee et al., 2020; Wang et al., 2021). We can further unlock this potential by starting with innovations in IDSPs.

### Emotional Interaction and Open Innovation in Industrial Design

IDSP is critical for addressing the lack of design knowledge in SMEs (Uskenbayeva et al., 2020; Lestantri et al., 2022; Liu J. et al., 2022). Industrial manufacturers are increasingly developing digital platforms to openly innovate their designs in a B2B environment in order to improve their products’ competitiveness (Brink, 2017; Costa et al., 2020; Rossi et al., 2022). This innovation, fueled by emotional interactions with customers *via* digital platforms, creates new business opportunities for industrial manufacturing companies (Jovanovic et al., 2021). These opportunities are intended to increase user satisfaction while also demonstrating how digital technologies can help businesses with their innovation efforts (Chowdhury et al., 2021). Therefore, it expands the scope of digital service innovation while also paving the way for traditional manufacturing to be transformed (Pyatt-Downes and Kane, 2019b; Retolaza et al., 2021). This has resulted in a shift away from product-centric business and toward more flexible and emotional interactions with users, allowing companies to gain market competitiveness through open (design) innovation (Sharma et al., 2021; Tseng et al., 2021).

Greening businesses through open innovation design is currently proving to be an effective way to increase market competitiveness (Retolaza et al., 2021; Ante et al., 2022). The free development and emotional interaction between users and product designers result in a new circular business model during the green transformation process (Zuljevic and Huybrechts, 2021; Wang Y. et al., 2022). This business model defines IDSPs, which are crucial in closing knowledge gaps and increasing innovation efficiency (Matos et al., 2019; Dahmani et al., 2021; Dokter et al., 2021). IDSP also automates the design-to-cost process, allowing design teams to manage any project based on the cost of each phase and component (Liu Z. et al., 2022). It effectively improves design market translation and increases the efficiency with which corporate innovations are commercialized (Camburn and Wood, 2018).

### The Influence of Emotional Interaction on the Marketization of Industrial Design

The most pressing issue confronting industrial design is rapid market response, but traditional user research is time-consuming and costly, severely limiting industrial design’s market sensitivity (Pyatt-Downes and Kane, 2019a). One effective way to address market sensitivity is to increase emotional interaction with users throughout the design process (Rosenzweig, 2015; Munirathinam, 2020). The industrial design service platform, on the other hand, creates objective conditions for emotional interactions between researchers (designers) and end users (Jovanovic et al., 2021; Zhang X. et al., 2022). Platform participants effectively connect industrial design to the market through real-time interaction in the community (Lee and Cassidy, 2007). This service platform, in the form of an online community, increases the efficiency of design commercialization by feeding design into market performance *via* emotional interaction with users. By coordinating various information and resources, designers are able to deliver innovative designs through emotional interactions to provide users with a sense of experience (surprise) that exceeds expectations, gaining market competitiveness (Rodríguez Ramírez, 2014; Becattini et al., 2020).

Instead of serving as a design tool for solving artistic or technical problems, IDSPs seek to systematize the design process (Nordin, 2018; Aldoy and Andrew Evans, 2021). As the number of digital natives increases and the range of digital design tools/media expands, the groundwork for a fully digital industrial design process is being laid (Pauliuk et al., 2022; Urgo et al., 2022). Using only the perceptual thinking and creativity of industrial designers, or exaggerating the role of quantitative data in the field of emotional cognition, on the other hand (Venturini, 2022), is a more one-sided approach to industrial design (Xiao and Cheng, 2020). Through emotional interaction with the target user, an industrial design service platform can effectively bridge this contradiction (Schneorson et al., 2019). Consequently, effective emotional interaction with users *via* an industrial design service platform is a powerful way to market design and integrate entrepreneurship into design practice.

## Research Design

### Research Methods

**Figure 1:**
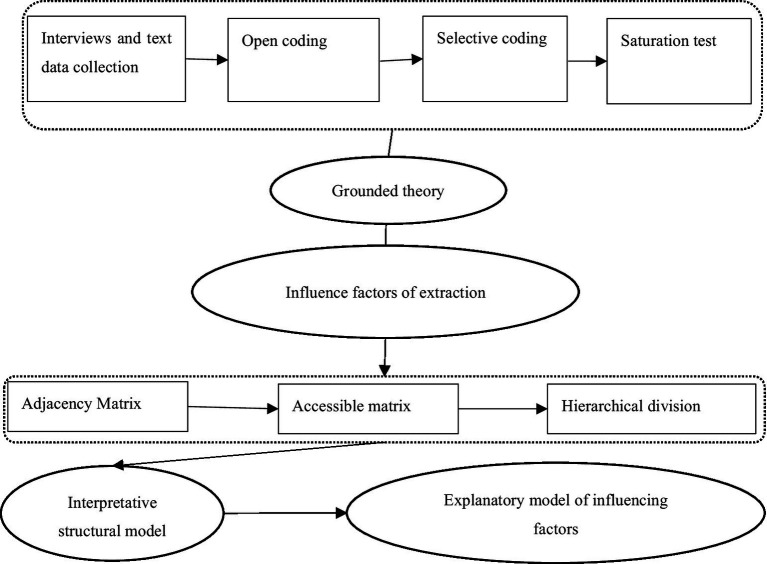
Research flow chart.

In this paper, the influencing factors of the industrial service platform are investigated using a combination of grounded theory and an explanatory structural model, as illustrated in Figure 1. Grounded theory is the primary method of preliminary qualitative research because the study’s sample data consist primarily of interviews and second-hand text data (Goulding, 2001). Since Glaser and Strauss proposed it in 1967, ground theory has been widely used in an inductive top-down qualitative research process to develop a specific phenomenon or thing and induce grounded theory (Soni, 2015). The coding of text data is central to this procedure. There are three steps for extracting samples from complex original data: open coding, selective coding, and saturation testing.

Following the principle of continuous comparative analysis, concepts and categories are summarized and refined from the original data until the theory is saturated and the theoretical model is formed. In terms of methodology, grounded theory, which is based on text mining method research based on the limitations of existing text materials, goes beyond the text data abstract theoretical concept, in order to better complete the hierarchy theory of inductive process, is immature for the analysis of industrial design service platform for the related influencing factors of the building theory of research (Zhang, 2021). This paper combined structural model interpretation with grounded theory abstract industrial design service platform to analyze the internal relationship between different factors in the analysis of the relationship between the factors related to the industrial design service platform. The interpretative structural model (ISM) is capable of decomposing complex and muddled system unit relationships into clear, multi-level, and hierarchical structural forms (Warfield, 1973).

### Data Source

This study collects and analyzes data using a method that combines first-hand interview data with second-hand case data to ensure the reliability and validity of the data obtained. To collect primary data, this paper relies heavily on semi-structured in-depth interviews. Among those interviewed were 10 industrial designers, 30 industrial design students (15 undergraduates and 15 master’s students), and 10 manufacturing practitioners (aged 22–45, 33 males and 27 females). The interviewees in this paper are highly involved in industrial design, and the interviews are mostly conducted in person with a video connection, lasting 15–30 min. The following questions are included in this paper’s formal interview outline: Personal information (name, gender, age, and occupation); Have you ever heard of or used a platform for industrial design services (website or mobile application)? What data and issues should I be aware of during the usage process? What difficulties did you encounter during the application process? Have you learned anything from these service platforms? What improvements do you think could be made to these platforms?

The interview outline served as a guide during the actual interview, the interview questions were framed in the context of the entire industrial design chain, and the interviewees were guided step by step through the process of expressing their opinions based on their responses (Xu et al., 2021). To overcome the issue of researchers’ mind-set suspension, the interview data were sorted and coded multiple times, and consensus was reached through a retrospective interview on ambiguities. Furthermore, based on the theoretical saturation test requirements, 80 percent of interview records (i.e., the first 40 interview records) were chosen for coding, with the remaining 10 used for saturation testing.

The triangulation method is used in this paper to collect case data. The data are primarily derived from 29 IDSPs, and the content related to platform requirements is extracted and summarized into text form, providing the original data for the grounded theory coding analysis process as well as basic support for the overall demand model study. The 29 industrial design service platform contains 19 regional industrial cloud (Beijing Industrial cloud, Deyang equipment manufacturing industrial cloud, Baoji industrial cloud platform, Changzhou intelligent manufacturing industrial cloud, Sanjiang Industrial cloud, Haixi industrial cloud platform, Fu’an (motor) industrial cloud, Guilin industrial cloud, Nanning industrial cloud, Jining industrial cloud, Xiamen Industrial cloud, Heilongjiang industrial enterprise public service platform, Dalian Industrial cloud, Zhucheng industrial cloud Inner Mongolia network coordinated manufacturing platform, Shaanxi digital manufacturing cloud service platform, Weihai enterprise public service cloud platform, Weifang industrial cloud, and Wenshang industrial cloud) and 10 industry industrial cloud (Tool housekeeper, valve cloud, excellent activity, D9X, Precision mold network, Yinzhou network, Tianhe mold cloud, T3 incubator, Earth energy cloud, and Linfen foundry industry cloud platform). Through the above multi-channel and multi-type data sources, the validity of research results is guaranteed.

### Data Encoding

#### Open Coding

The process of “crumpling” and “breaking up” raw data and redefining concept labels in order to decompose and conceptually categorize it is known as open coding (Zhang, 2021). To ensure objectivity, the code uses interviews and description statements from the industrial design service platform to conduct primary conceptualization and eliminate redundant original concepts, and it extracts higher-level categories based on logical relations to achieve data categorization (Xu et al., 2021; Aemmi et al., 2022). We have been constantly revising and checking with experts with associate professors and above titles in the industrial design major, repeatedly demonstrating the logical relationships between them, and have finally obtained 188 concepts and 36 categories, which are only partially listed in the table due to length (Table 1).

**Table 1:** Open coding categorization (partial examples).

Original statement P	Initial concept a	Category change A
P1: Pay more attention to frontier data and information, such as technology, materials, and design creativity, among other things. P2: Limited employment options, insufficient design market research, and high demand for professional skills. I’d like to get a reputable professional certification.	a1 Frontier discipline; a2 Shared; a3 Design research; a4 Training; a5 Certification;	A1 Industry development; A2 Design survey; A3 Skill requirements; A4 Certification and training;
P3: There are few related platforms to find design materials and inspirations, and they need to be charged. P4: Without standards, it is difficult to define the quality of design. P5: Undertake design orders.	a7 Design materials and inspiration; a8 Paid services; a9 Evaluation criteria; a10 Design market;	A5 Source of creation; A6 Evaluation system; A7 Design barrier; A8 Design transformation;
P6: Related competition, hope that the winner can be connected with the enterprise and put into production. P7: I hope relevant platforms can enter reliable enterprise recruitment to help solve the employment problem.	a11 Design competition; a12 Disconnection between design and production; a13 Employment;	A9 Design talents; A10 Design and production matching;
P8: Insufficient learning and training of employees in the process of manufacturing. P9: Insufficient capacity, single equipment, and no replacement equipment. P10: It is difficult to grasp the real needs of users, which requires coordination by platforms or departments.	a14 Continuous learning; a15 Equipment maintenance; a16 User requirements; a17 Design collaboration;	A11 Industry-university-research institute disconnect; A12 Design upgrade; A13 Supply and demand matching; A14 Design efficiency; A15 Design collaboration;
P11: regional industrial cloud mainly provides customized marketing, R & D and design, and intelligent production. Products and services, digital strategy consulting, enterprise innovation construction consulting, big data innovation development and application, design and manufacturing feasibility consulting P12: Research on industrial design strategy and trend, public service of design resources, regional industrial upgrading, docking of large projects, high-tech industrialization and innovation education. P13: Integrate multiple resources, provide integrated solutions and promote industrial innovation based on the needs of enterprises, government and education for innovation and design	a18 Industrial design cloud; a19 Public service of design resources; a20 Design and industrial upgrading; a21 Design item matching; a22 Technological innovation and design; a23 Design education innovation; a24 Product design and service; a25 Design resource supply; a26 Industrial Design Frontier; a27 Industrial design strategy; a28 Industrial design trends; a29 Itegrated solution;	A16 Regional industrial cloud; A16 Public services; A15 Design education; A16 Design resource integration; A17 Innovative design; A18 Design supporting services; A19 Design entrepreneurship and team; A20 Design problem integration; A21 Design frontier; A22 Design internationalization; A23 Design supporting services; A24 Open communication platform;
P14: Innovation and entrepreneurship team, design team and manufacturing product R&D department provide relevant services P15: International design supporting services, to provide excellent works exhibition, for academic exchange, regional industry information, venue rental, resource replacement services.	a30 Design entrepreneurship; a31 Design internationalization; a32 Supporting services; a33 Design team; a34 Exchange; a35 Resource matching;	
P16: Industrial cloud mainly provides design consultation, technical support and docking, event exchange and brand promotion P17: In-depth thematic, interactive design, exhibition competition, Design circle and cluster, industrial park, release platform, training, patent/law, material recommendation, Award works, design materials, creative gallery	a36 Industry industry cloud; a37 Information consultation; a38 Brand promotion; a39 Industrial docking; a40 Design marketing; a41 Design cluster; a42 Depth analysis; a43 Design recommendation; a44 Race; a45Publishing platform; a46 Works Library; a47 Patent/Law; a48 Design materials;	A25 Industry cloud; A26 Resource allocation; A27 Technical support; A28 Promotion; A29 Industrial docking; A30 Design competition; A31 Patent protection; A32 Legal support; A33 Interaction design; A34 Database;
P18 Product image, series of product PI design, project promotion, benchmark industrial design, product design, UI design, mechanical design, structural design, circuit design, processing, mold manufacturing, non-standard equipment design in a complete industrial chain integrated design.	a49 Creative gallery; a50 Image design; a51 Product design; a52 Brand planning; a53 Interface design; a54 Mechanical design; a55 Structure design; a56 Design and processing; a57 Model design; a58 Mold manufacturing; a59 Non-standard design	A35 Product design and brand planning; A36 Mechanical structure design; A37 Mold design and manufacturing; A38 Foundation design; A40 Non-standard design (custom)

The interview results of the 50 subjects were sorted, answers that did not fit the purpose of the interview were removed, and the original text with the platform requirements vision was retained. Furthermore, the network information of 29 IDSPs is sorted out in relation to the platform’s design services, which is represented by P as the original data. Ten interview samples and five platform samples were set aside to be analyzed later for theoretical saturation.

#### Axial Coding

Spindle coding is based on open coding in order to hide the deep logical relationships between the main categories and then dig out the main categories. Based on a thorough examination of the industrial design service platform’s demand orientation, this paper abstracts the 40 main categories based on the logic and operation of the main axis coding, and finally extracts 16 main categories. The “design demand” and “design supply” of the main category are extracted from the input of design-related knowledge, skills, resources, and information of the sub-category, reflecting the intuitive needs of users, according to coding rules. As shown in Table 2.

**Table 2:** Categorization of spindle codes.

Category (A)	Sub-category (AA)	Main category (AAA)
A1 Industry development; A2 Design survey; A3 Skill requirements; A4 Certification and training; A5 Source of creation; A6 Evaluation system; A7 Design barrier; A8 Design transformation; A9 Design talents; A10 Design and production matching; A11 Industry-university-research institute disconnect; A12 Design upgrade; A13 Supply and demand matching; A14 Design efficiency; A15 Design collaboration; A16 Regional industrial cloud; A16 Public services; A15 Design education; A16 Design resource integration; A17 Innovative design; A18 Design supporting services; A19 Design entrepreneurship and team; A20 Design problem integration; A21 Design frontier; A22 Design internationalization; A23 Design supporting services; A24 Open communication platform; A25 Industry cloud; A26 Resource allocation; A27 Technical support; A28 Promotion; A29 Industrial docking; A30 Design competition; A31 Patent protection; A32 Legal support; A33 Interaction design; A34 Database; A35 Product design and brand planning; A36 Mechanical structure design; A37 Mold design and manufacturing; A38 Foundation design; A40 Non-standard design (custom)	AA1 Industry trend; AA2 Design requirements; AA3 Design supply; AA4 Education disconnects; AA5 Supply and demand disjointed; AA6 Design update; AA7 Design promotion; AA8 Design collaboration; AA9 Design innovation; AA10 Resource integration; AA11 Information exchange; AA12 Skill output; AA13 International standards; AA14 Design transaction; AA15 Design matching; AA16 Intellectual property rights; AA17 Basic design; AA18 Design cloud; AA19 Policies and regulations; AA20 Technical foundation; AA21 Equipment foundation; AA22 Data support; AA23 Design matching; AA27 Non-standard design; AA28 Products and brands	AAA1 Frontier trend; AAA2 Design supply and demand; AAA3 Industry, education and research; AAA4 Design efficiency; AAA5 Update and promotion; AAA6 Collaborative innovation; AAA7 Design resources; AAA8 Design marketization; AAA9 Intellectual property rights; AAA10 Foundation design; AAA11 Design internationalization; AAA12 Design digitization; AAA13 Policies and regulations; AAA14 Design creativity library; AAA15 Supporting services; AAA16 Personality design

#### Selective Coding

Selective coding is a system analysis process that establishes the core category of the entire demand and the coupling relationship between the other categories, as well as the logic and correlation between 16 main categories based on causality, transmission mechanism, and further implementation root path—the model results, system to establish the core category of the entire demand and the coupling relationship between the other categories, and further clear the inner m. From thinking and analyzing the connotation and mutual relationship between the main categories, four core categories of demand type, design matching, design integration, and design transformation are derived, and these four categories are used to organize other categories. The entire selection code is shown in Table 3. Design matching runs through the core category of activities as a thread through the entire requirements. The core category begins with demand type as a causal condition, and design integration is the intermediate condition that promotes design transformation.

**Table 3:** Selective coding.

Typical paradigm	Main categories	Core category
Causal relationship	Frontier trend; Update and promotion; The character designs	Requirement type
Transmission mechanism	Design supply and demand; Supporting services	Design match
Realize the path	Policies and regulations; Production; Design internationalization; Design digitization; Design resources; Design efficiency; Collaborative innovation	Design integration
Results	Design marketization; Intellectual property rights; Foundation design; Design creativity library	Design transformation

#### Theoretical Saturation Test

To ensure the reliability of the final demand analysis model, theoretical saturation must be tested. According to Gleiser and Strauss, theoretical saturation occurs when an analyst can no longer obtain data from the data to further develop the characteristics of a category (Zhang, 2021). This study discovered that no new concepts or categories were generated after coding the last 10 interview texts and design service texts of five industrial design innovation service platforms, indicating that the platform demand model constructed through the coding text process is essentially saturated.

## Construction of Explanatory Structural Model for Influencing Factors of Industrial Design Service Platform

### The Adjacency Matrix of Influencing Factors of Industrial Design Service Platform Is Established

This article is based on the concept of grounded theory to analyze the abstract, summarize the influence factors of industrial design service platform into three categories: the dominant factors, respectively, the government’s leading, leading, and enterprise leading colleges and universities, and analyze the industrial design service platform in the system the relationship between various influencing factors through the establishment of adjacency matrix. There are six factors in the government-led type, six factors in the enterprise-led type, and four factors in the college-led type. Furthermore, the Delphi method is used to consult experts above associate professors in the field of industrial design and industry personnel who have worked in the field for more than 3 years in order to construct a 16 × 16 adjacency matrix. As shown in Formula (1), it is denoted as 1 if there is a mutual influence relationship between factors and columns, and 0 if there is no mutual influence relationship (1). Table 4 depicts the factors as columns and columns.


(1) $$M=\left[\begin{array}{l} 0000000000000000\\ 1000110011000111\\ 1001001000110000\\ 1110000100011000\\ 1010000001011100\\ 1100100110000000\\ 1000010001010011\\ 1001101000101000\\ 1010001000000010\\ 1000100010000010\\ 1100000100100000\\ 1000010001010111\\ 1011000000001101\\ 1000001010000000\\ 1000100000100000\\ 1100001001000010 \end{array}\right]$$

**Table 4:** Variables represented by each column in the matrix.

Column	Variable	Column	Variable
1	Y——Influencing factors of industrial design service platform		
2	A1——Frontier trend	10	A9——Intellectual property right
3	A2——Design supply and demand	11	A10——Foundation design
4	A3——Industry-university research	12	A11——Design internationalization
5	A4——Design efficiency	13	A12——Design digitization
6	A5——Update and promotion	14	A13——Policies and regulations
7	A6——Collaborative innovation	15	A14——Design creativity Library
8	A7——Design resources	16	A15——Supporting services
9	A8——Design Marketization	17	A16——Personality design

### Establish Reachability Matrix

The reachable matrix n is obtained by performing a Boolean operation on the adjacency matrix and the identity matrix. A matrix can be used to express the degree to which each node in a directed connection graph can reach a given length of path (Huang et al., 2014; Isla and Teknomo, 2016). The specific steps, according to Huang et al. (2014), are Boolean summation of adjacency matrix M and identity matrix, followed by iterative operations until Equation (2) is established as:


(2) $${\left(M+I\right)}^{n-1}\neq {\left(M+I\right)}^{n}={\left(M+I\right)}^{n+1}$$

When Formula (2) is established, the reachable matrix is as:


(3) $$N={\left(M+I\right)}^{n}$$

The reachable matrix in this paper is shown in Equation (4), which can be expressed as the interaction relationship and transitivity between factors A1 and A16.


(4) $$N=\;\;\left[\begin{array}{l} 1000000000000000\\ 1100010101010100\\ 1010100010100010\\ 1011101000101010\\ 1001101000000101\\ 1100110001010001\\ 1010011110000000\\ 1000101100010101\\ 1000010110010000\\ 1010101001100000\\ 1000000100100100\\ 1001000010011010\\ 1000010001001001\\ 1000000000101101\\ 1000001000010010\\ 1000000100001001 \end{array}\right]$$

### Reduce the Matrix and Layer

The relationship between the influencing factors of the operation of the industrial design service platform can be further determined by dividing the hierarchy of the accessibility matrix N. The set of elements corresponding to all columns in the ith row of the reachable matrix with matrix element 1 is known as the reachable set A. The antecedent set B is defined as the set of elements corresponding to all rows with matrix element 1, defines the reachable and antecedent sets’ common set C = A∩B. When C = A for a given element is satisfied, the element can be extracted hierarchically. After the element is extracted, the rows and columns of the extracted element pairs are removed from the reachable matrix, and the process is repeated for the next level of element extraction. Repeat the element extraction procedure, deleting rows and columns from the reachable matrix until all elements are extracted. Using the process described above, the reachable matrix is extracted, and five levels are obtained as: L1 = {Y}; L2 = {A13, A15}; L3 = {A1,A5,A6，A11,A12,A16}; L4 = {A3,A4,A7,A9,A14}; L5 = {A2,A8,A10}.

### Structural Model of Influencing Factors of Industrial Design Service Platform

**Figure 2:**
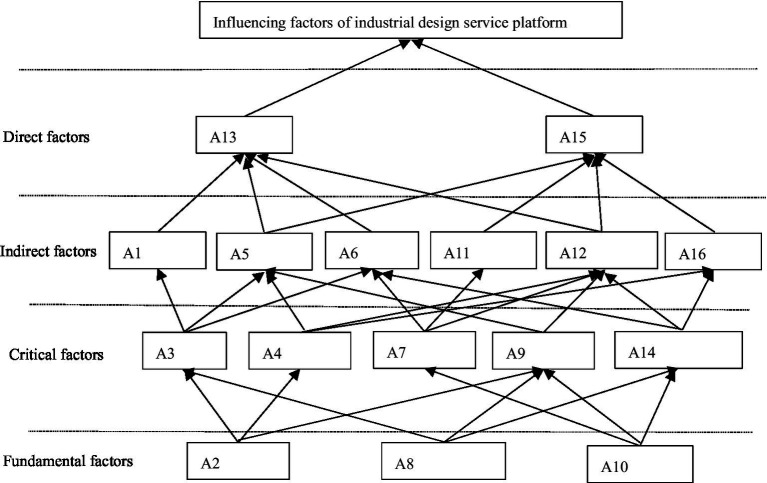
ISM model of influencing factors of industrial design service platform.

The ISM model of industrial design service platform as shown in Figure 2 is drawn based on the level extraction of elements, and then, the association is established according to the adjacent level and the same level of accessible set connection, and finally, the explanatory structural model of influencing factors of industrial design service platform is obtained.

### Results Analysis

According to the ISM diagram of influencing factors of industrial design service platform in Figure 2, the factors influencing industrial design service platform have a 4-level and multi-layer progressive structure, and the relationships between direct factors, indirect factors, key factors, and basic factors are also determined.

Basic factors are those that make up or influence the formation or development of things and are unaffected by other variables. The most fundamental driving forces influencing the industrial design service platform are the government and businesses. The role of government leadership in the development of the industrial design service platform has an impact on the platform’s development direction and social potential. Interviewees frequently express high confidence in government-led projects, which is primarily reflected in their belief that government-led projects will provide adequate financial support for basic design and material R&D, improve the trading market, and increase the design market conversion rate. Simultaneously, as a result of the government’s ongoing support, design intellectual property rights will be strengthened, boosting designers’ confidence. Furthermore, because of the government’s hegemony, policies can aid in the construction of an industrial design service platform, allowing the platform to gain a foothold in the market by relying on policy advantages during the initial stages of construction. At the basic factor level, government-led scientific research units frequently conduct basic design and material research and development. Such design and research and development are frequently expensive and time-consuming, making them difficult for a single company to bear. China’s design trading market, on the other hand, is far from perfect. As a result of a blind policy area, market subjects and individuals frequently conduct separate private transactions, making it difficult to protect both parties’ legal rights. As a result, protecting design intellectual property rights frequently takes a long time and costs a lot of money while producing no results.

Among the factors dominated by businesses, respondents frequently place a high value on market sensitivity. Because the majority of Chinese universities’ design education is severely disconnected from market demand, colleges and universities face a dual dilemma of design demand input and output difficulties. The primary causes of this situation are as follows: First, the domestic design market is still in its infancy, with corresponding market vitality and demand not fully developed; second, in the process of developing talent, colleges and universities place an overabundance of emphasis on theoretical knowledge study while ignoring the role of practice. Third, design education remains a niche subject in China, and society lacks a fundamental understanding of design. Design development in China is still in its early stages, and a comprehensive talent training system has yet to emerge. Therefore, the input and output of design requirements in the influence factors of the enterprise-led industrial design service platform are the fundamental links to realizing the improvement of industrial design innovation transformation efficiency.

Among the key factors, the leading factors of universities occupy the primary position, with the introduction of advanced equipment playing a critical role in the practical operation ability of talents training, and numerous creative ideas are frequently limited to the conceptual level and difficult to achieve due to equipment limitations. The introduction of advanced equipment, on the other hand, frequently necessitates a significant financial investment, making it difficult for the vast majority of colleges and universities to invest in the design discipline. Only a few colleges and universities are capable of doing so, and the talents trained by these colleges and universities are unable to meet the market’s diverse needs, posing a significant barrier to the growth of the industrial design industry. In terms of design outcomes, the two levels of differentiation between universities are more obvious: excellent colleges and universities have numerous accomplishments or cannot be transformed, whereas ordinary colleges and universities have few accomplishments. In terms of design competitions, project implementation, design research, and design training, for example, the differences between colleges and universities are vast. General colleges and universities design competitions and project resources, even for related resources, can be difficult to have enough support for its further development, the basis of good colleges and universities is further monopoly resources, which leads to resource waste formation.

Enterprises clearly dominate indirect factors, and only enterprises can realize all of the elements. China does not yet have a fully developed industrial chain in terms of design information and consulting, with only sporadic development in major developed urban agglomerations, and the vast domestic market remains untapped. It also demonstrates the convergence of major cities in terms of design exhibition, recommendation, and design forum, whereas design exhibition and forum have devolved into mere formalities and concepts.

## Discussion

### Conclusion

Based on grounded theory, this paper combines qualitative and quantitative research to examine the factors influencing demand for IDSPs in the context of industry-university-research collaborative innovation, and it summarizes the elements that must be considered when developing an industrial design service platform. As China’s economy transitions into an era of innovation-driven high-quality development, the industrial design service platform based on collaborative innovation between industry, universities, and research will become a powerful tool for businesses to innovate, develop, and promote high-quality economic growth. However, because the industrial design service platform and even industrial design are still relatively new in China, much of the research on the industrial design service platform is still in its early stages, with no comprehensive research theory or framework in place. Hence, in this paper, grounded theory is used to extract and define the influence variables of an industrial design service platform from a large amount of empirical text data. Furthermore, an interpretive structural model is used to stratify the influencing factors of the industrial design service platform, and the influencing factors model is built with the objective context in mind.

### Theoretical Contribution

The existing research contributions discussed in this paper are as follows: (1) The “government leading, leading, enterprise leading colleges and universities” and other three types of industrial design and the service platform’s operation mode are presented, comprehensively summarizing the industrial design service platform of variable scope, based on the production under the collaborative innovation influence factors of industrial design service platform. (2) Currently, there is no systematic study of the interaction and mechanism of influencing factors of an industrial design service platform. This paper abstracts these factors from a large amount of existing text data to form specific variable concepts and then discusses the relationship between variables using an explanatory structural model. To explain the influencing factors and mechanisms of the development of an industrial design service platform, a deeper theoretical level is used.

The study’s findings provide a theoretical foundation for improving the industrial design service platform. On the one hand, this article will concentrate on the production area of industrial design service platform variables against the backdrop of collaborative demand influence factors and will present the factors influencing industrial design service platform causality—transmission mechanism, paths, and the results of analysis paradigm, the more comprehensive combing the influence the demand for industrial service platform variables. Previous research, on the other hand, has not done a thorough elaboration and analysis of the relationship between various influencing factors. This paper summarizes the industrial design service platform demand influence factors on the basis of furthering the relationship between the various factors for the detailed comb, from a deeper understanding of the cause of all the factors and their mechanism.

### Practical Contribution

Not only do our findings provide a theoretical framework for understanding the mechanisms underlying various influencing factors of industrial design service platform demand, but they also open up a new research avenue for future industrial design service platform demand research and analysis. It also serves as a practical guide for the efficient construction and operation of an industrial design service platform. The level of construction of the industrial design service platform, first and foremost, cannot be overlooked. In the future, more emphasis should be placed on meeting the personalized needs of users in the online (digital) and cross-border (international) environments of user demand services. Furthermore, cultivating users’ correct understanding of industrial design will help users gain a better understanding of their own needs and locate the works that they truly require. Second, government-led factors have a direct impact on the development of an industrial design service platform, and the government’s current disregard for industrial design exacerbates the design market’s supply-demand imbalance, which has become a major impediment to industrial upgrading. Consequently, the government should keep improving relevant systems and regulations, as well as actively building and guiding a professional industrial design service platform. Finally, the supply and demand for design, the level of marketization, and the three basic design elements are the basic elements of influencing factors of demand for an industrial design service platform. As a result, the government should actively guide and cultivate the enhancement of fundamental design ability while also promoting the establishment of an industrial design service platform.

The direct factors are clearly dominated by the government. The government is usually in charge of getting things done, whether it is policy guidance or infrastructure. Policy guidance is currently the most important source of support for the development of the domestic industrial design service platform, which is still in a semi-market state. The fact that units and organizations with strong design capabilities frequently have a strong government background, or are even directly affiliated with government departments, reflects design docking and support. Consequently, businesses frequently find it more convenient to obtain design support and docking with design firms only with government approval. Simultaneously, the construction of supporting facilities related to design is often difficult to achieve by a single enterprise, necessitating early government investment to create a good environment for further progress.

### Limitations and Future Research

The study’s findings and flaws serve as a starting point for future research. To begin, the research data sources used in this paper are limited, with only online questionnaires and offline interviews used, resulting in a sample that is not sufficiently representative. Large-scale data collection, however, is difficult due to the COVID-19 epidemic and a lack of research funds. Meanwhile, our research is limited to 29 IDSPs, including 19 regional industrial clouds and 10 industrial clouds, indicating that China has yet to establish a large-scale industrial design cloud, which will necessitate future development. Second, there are currently few studies on IDSPs, and the research presented in this paper is only an exploratory study of the factors influencing demand. This is only a small sample of research findings; larger-scale and more in-depth testing of the practice are required.

As the digital economy and economic globalization continue to develop, China is becoming increasingly aware of the importance of industrial design and innovative industrial design services, as well as the impact of industrial design on the construction of a new industrial ecosystem in the fourth Industrial Revolution (Industry 4.0). This study can help researchers gain experience, make breakthroughs in future research, and significantly contribute to the transformation and upgrading of traditional manufacturing industries.

## Data Availability Statement

The original contributions presented in the study are included in the article/supplementary material; further inquiries can be directed to the corresponding authors.

## Ethics Statement

Ethical review and approval were not required for the study on human participants in accordance with the local legislation and institutional requirements. Written informed consent from the patients/participants or patients/participants legal guardian/next of kin was not required to participate in this study in accordance with the national legislation and the institutional requirements.

## Author Contributions

CZ: conceptualization, methodology, software, writing - original draft preparation; QY: validation, formal analysis, investigation, resources, supervision, funding acquisition, supervision; LT: data curation, writing — review and editing; visualization. RZ: writing — original draft preparation, data collection. All authors have read and agreed to the published version of the manuscript.

## Conflict of Interest

The authors declare that the research was conducted in the absence of any commercial or financial relationships that could be construed as a potential conflict of interest.

## Publisher’s Note

All claims expressed in this article are solely those of the authors and do not necessarily represent those of their affiliated organizations, or those of the publisher, the editors and the reviewers. Any product that may be evaluated in this article, or claim that may be made by its manufacturer, is not guaranteed or endorsed by the publisher.

## References

[ref1] Aemmi S. Z. Mohammadi E. Fereidooni-Moghadam M. Zarea K. Boostani H. (2022). Sleep management experiences of shift-working nurses: a grounded theory study. Collegian. doi: 10.1016/j.colegn.2021.11.005

[ref2] Aldoy N. Andrew Evans M. (2021). An investigation into a digital strategy for industrial design education. Int. J. Art Des. Educ. 40, 283–302. doi: 10.1111/jade.12334

[ref3] An Y. Qin F. Sun D. Wu H. (2020). A multi-facets ontology matching approach for generating PLC domain knowledge graphs. IFAC-PapersOnLine 53, 10929–10934. doi: 10.1016/j.ifacol.2020.12.2834

[ref4] Ante L. Fischer C. Strehle E. (2022). A bibliometric review of research on digital identity: research streams, influential works and future research paths. J. Manuf. Syst. 62, 523–538. doi: 10.1016/j.jmsy.2022.01.005

[ref5] Bartliff Z. Kim Y. Hopfgartner F. Baxter G. (2020). Leveraging digital forensics and data exploration to understand the creative work of a filmmaker: a case study of Stephen Dwoskin’s digital archive. Inf. Process. Manag. 57:102339. doi: 10.1016/j.ipm.2020.102339

[ref6] Becattini N. Borgianni Y. Cascini G. Rotini F. (2020). Investigating users’ reactions to surprising products. Des. Stud. 69:100946. doi: 10.1016/j.destud.2020.05.003

[ref7] Benitez J. Arenas A. Castillo A. Esteves J. (2022). Impact of digital leadership capability on innovation performance: the role of platform digitization capability. Inf. Manag. 59:103590. doi: 10.1016/j.im.2022.103590

[ref8] Brand G. Hendy L. Harrison R. (2015). Mining the gap! Fostering creativity and innovative thinking. Procedia Technol. 20, 79–84. doi: 10.1016/j.protcy.2015.07.014

[ref9] Brink T. (2017). B2B SME management of antecedents to the application of social media. Ind. Mark. Manag. 64, 57–65. doi: 10.1016/j.indmarman.2017.02.007

[ref10] Bu L. Zhang Y. Liu H. Yuan X. Guo J. Han S. (2021). An IIoT-driven and AI-enabled framework for smart manufacturing system based on three-terminal collaborative platform. Adv. Eng. Inform. 50:101370. doi: 10.1016/j.aei.2021.101370

[ref11] Camburn B. Wood K. (2018). Principles of maker and DIY fabrication: enabling design prototypes at low cost. Des. Stud. 58, 63–88. doi: 10.1016/j.destud.2018.04.002

[ref12] Castelo-Branco R. Caetano I. Leitão A. (2022). Digital representation methods: the case of algorithmic design. Front. Archit. Res. doi: 10.1016/j.foar.2021.12.008

[ref13] Cheng V. M. Y. (2019). Developing individual creativity for environmental sustainability: using an everyday theme in higher education. Think. Skills Creat. 33:100567. doi: 10.1016/j.tsc.2019.05.001

[ref14] Chowdhury S. Åkesson M. Thomsen M. (2021). Service innovation in digitalized product platforms: an illustration of the implications of generativity on remote diagnostics of public transport buses. Technol. Soc. 65:101589. doi: 10.1016/j.techsoc.2021.101589

[ref15] Christou I. T. Kefalakis N. Soldatos J. K. Despotopoulou A.-M. (2022). End-to-end industrial IoT platform for quality 4.0 applications. Comput. Ind. 137:103591. doi: 10.1016/j.compind.2021.103591

[ref16] Corazza G. E. Reiter-Palmon R. Beghetto R. A. Lubart T. (2021). Intelligence and creativity in the space-time continuum for education, business, and development. J. Creat. 31:100003. doi: 10.1016/j.yjoc.2021.100003

[ref17] Costa E. Soares A. L. de Sousa J. P. (2020). Industrial business associations improving the internationalisation of SMEs with digital platforms: a design science research approach. Int. J. Inf. Manag. 53:102070. doi: 10.1016/j.ijinfomgt.2020.102070

[ref18] da Silva Rodrigues C. K. (2021). Analyzing Blockchain integrated architectures for effective handling of IoT-ecosystem transactions. Comput. Netw. 201:108610. doi: 10.1016/j.comnet.2021.108610

[ref19] Dahmani N. Benhida K. Belhadi A. Kamble S. Elfezazi S. Jauhar S. K. (2021). Smart circular product design strategies towards eco-effective production systems: a lean eco-design industry 4.0 framework. J. Clean. Prod. 320:128847. doi: 10.1016/j.jclepro.2021.128847

[ref20] Dokter G. Thuvander L. Rahe U. (2021). How circular is current design practice? Investigating perspectives across industrial design and architecture in the transition towards a circular economy. Sustain. Prod. Consum. 26, 692–708. doi: 10.1016/j.spc.2020.12.032

[ref21] Dumitrache I. Caramihai S. I. Sacala I. S. Moisescu M. A. Popescu D. C. (2020). Future Enterprise as an intelligent cyber-physical system. IFAC-PapersOnLine 53, 10873–10878. doi: 10.1016/j.ifacol.2020.12.2817

[ref22] Espinoza Pérez A. T. Rossit D. A. Tohmé F. Vásquez Ó. C. (2022). Mass customized/personalized manufacturing in industry 4.0 and blockchain: research challenges, main problems, and the design of an information architecture. Inf. Fusion 79, 44–57. doi: 10.1016/j.inffus.2021.09.021

[ref23] FuJun W. Zhou Y. Ying Gang O. Xiang Jun Z. Jie Li D. (2018). “Government-industry-university-research-promotion” collaborative innovation mechanism construction to promote the development of agricultural machinery technology. IFAC-PapersOnLine 51, 552–559. doi: 10.1016/j.ifacol.2018.08.147

[ref24] Goulding C. (2001). Grounded theory: A magical formula or a potential nightmare. Mark. Rev. 2, 21–33. doi: 10.1362/1469347012569409

[ref25] Hao R. Cheng Y. Zhang Y. Tao F. (2021). Manufacturing service supply-demand optimization with dual diversities for industrial internet platforms. Comput. Ind. Eng. 156:107237. doi: 10.1016/j.cie.2021.107237

[ref26] He G. Dang Y. Zhou L. Dai Y. Que Y. Ji X. (2020). Architecture model proposal of innovative intelligent manufacturing in the chemical industry based on multi-scale integration and key technologies. Comput. Chem. Eng. 141:106967. doi: 10.1016/j.compchemeng.2020.106967

[ref27] He L. Xue M. Gu B. (2020). Internet-of-things enabled supply chain planning and coordination with big data services: certain theoretic implications. Journal of Management Science and Engineering 5, 1–22. doi: 10.1016/j.jmse.2020.03.002

[ref28] Hsiao C.-H. Lee W.-P. (2021). OPIIoT: design and implementation of an open communication protocol platform for industrial internet of things. Internet Things 16:100441. doi: 10.1016/j.iot.2021.100441

[ref29] Huang B. Jiang R. Zhang G. (2014). Comments on “heuristic search for scheduling flexible manufacturing systems using lower bound reachability matrix”. Comput. Ind. Eng. 67, 235–236. doi: 10.1016/j.cie.2013.11.012

[ref30] Isla L. Teknomo K. (2016). Analysis of metro Manila road network robustness through reachability matrix. Procedia. Soc. Behav. Sci. 218, 141–151. doi: 10.1016/j.sbspro.2016.04.017

[ref31] Jianjia H. Gang L. Xiaojun T. Tingting L. (2021). Research on collaborative recommendation of dynamic medical services based on cloud platforms in the industrial interconnection environment. Technol. Forecast. Soc. Chang. 170:120895. doi: 10.1016/j.techfore.2021.120895

[ref32] Jovanovic M. Sjödin D. Parida V. (2021). Co-evolution of platform architecture, platform services, and platform governance: expanding the platform value of industrial digital platforms. Technovation:102218. doi: 10.1016/j.technovation.2020.102218

[ref33] Kabukcu E. (2015). Creativity process in innovation oriented entrepreneurship: the case of Vakko. Procedia. Soc. Behav. Sci. 195, 1321–1329. doi: 10.1016/j.sbspro.2015.06.307

[ref34] Kori G. S. Kakkasageri M. S. Manvi S. K. S. (2021). “Chapter 3—computational intelligent techniques for resource management schemes in wireless sensor networks,” in Recent Trends in Computational Intelligence Enabled Research. eds. Bhattacharyya S. Dutta P. Samanta D. Mukherjee A. Pan I. (Academic Press), 41–59.

[ref35] Lawson B. (2005). How Designers Think. 4th *Edn.* Routledge, Elsevier.

[ref36] Lee K. C. K. Cassidy T. (2007). Principles of design leadership for industrial design teams in Taiwan. Des. Stud. 28, 437–462. doi: 10.1016/j.destud.2006.11.007

[ref37] Lee J. Singh J. Azamfar M. Pandhare V. (2020). “Chapter 8—industrial AI and predictive analytics for smart manufacturing systems,” in Smart Manufacturing. eds. Soroush M. Baldea M. Edgar T. F. (Elsevier), 213–244.

[ref38] Lestantri I. D. Janom N. B. Aris R. S. Husni Y. (2022). The perceptions towards the digital sharing economy among SMEs: preliminary findings. Procedia Comput. Sci. 197, 82–91. doi: 10.1016/j.procs.2021.12.121

[ref39] Li P. Cheng Y. Tao F. (2020). Failures detection and cascading analysis of manufacturing services collaboration toward industrial internet platforms. J. Manuf. Syst. 57, 169–181. doi: 10.1016/j.jmsy.2020.08.012

[ref40] Liu Z. Sampaio P. Pishchulov G. Mehandjiev N. Cisneros-Cabrera S. Schirrmann A.. (2022). The architectural design and implementation of a digital platform for industry 4.0 SME collaboration. Comput. Ind. 138:103623. doi: 10.1016/j.compind.2022.103623

[ref41] Liu J. Zhou H. Chen F. Yu J. (2022). The coevolution of innovation ecosystems and the strategic growth paths of knowledge-intensive enterprises: the case of China’s integrated circuit design industry. J. Bus. Res. 144, 428–439. doi: 10.1016/j.jbusres.2022.02.008

[ref42] Mariani M. M. Nambisan S. (2021). Innovation analytics and digital innovation experimentation: the rise of research-driven online review platforms. Technol. Forecast. Soc. Chang. 172:121009. doi: 10.1016/j.techfore.2021.121009

[ref43] Matos D. Terroso M. Sampaio J. (2019). The growing path in search of an industrial design identity. Procedia CIRP 84, 353–356. doi: 10.1016/j.procir.2019.05.017

[ref44] Matthews B. Khan A. H. Snow S. Schlosser P. Salisbury I. Matthews S. (2021). How to do things with notes: The embodied socio-material performativity of sticky notes. Des. Stud. 76:101035. doi: 10.1016/j.destud.2021.101035

[ref45] Morgan J. Halton M. Qiao Y. Breslin J. G. (2021). Industry 4.0 smart reconfigurable manufacturing machines. J. Manuf. Syst. 59, 481–506. doi: 10.1016/j.jmsy.2021.03.001

[ref46] Mourtzis D. Angelopoulos J. Panopoulos N. (2021). A survey of digital B2B platforms and marketplaces for purchasing industrial product service systems: a conceptual framework. Procedia CIRP 97, 331–336. doi: 10.1016/j.procir.2020.05.246

[ref47] Munirathinam S. (2020). “Chapter 6: Industry 4.0: Industrial Internet of Things (IIOT),” in Advances in Computers. eds. Raj P. Evangeline P., Vol. 117 (Elsevier), 129–164.

[ref48] Nain G. Pattanaik K. K. Sharma G. K. (2022). Towards edge computing in intelligent manufacturing: past, present and future. J. Manuf. Syst. 62, 588–611. doi: 10.1016/j.jmsy.2022.01.010

[ref49] Nordin A. (2018). Challenges in the industrial implementation of generative design systems: an exploratory study. AI EDAM 32, 16–31. doi: 10.1017/S0890060416000536

[ref50] Ogundoyin S. O. Kamil I. A. (2021). Optimization techniques and applications in fog computing: An exhaustive survey. Swarm Evol. Comput. 66:100937. doi: 10.1016/j.swevo.2021.100937

[ref51] Oliva F. L. Teberga P. M. F. Testi L. I. O. Kotabe M. Giudice M. D. Kelle P.. (2022). Risks and critical success factors in the internationalization of born global startups of industry 4.0: a social, environmental, economic, and institutional analysis. Technol. Forecast. Soc. Chang. 175:121346. doi: 10.1016/j.techfore.2021.121346

[ref52] Onsman A. (2016). Assessing creativity in a ‘new generation’ architecture degree. Think. Skills Creat. 19, 210–218. doi: 10.1016/j.tsc.2015.07.001

[ref53] Otto T. Deger J. Marcus G. E. (2021). Ethnography and exhibition design: insights from the Moesgaard inaugural. Des. Stud. 74:100989. doi: 10.1016/j.destud.2020.100989

[ref54] Paay J. Kuys B. Taffe S. (2021). Innovating product design through university-industry collaboration: codesigning a bushfire rated skylight. Des. Stud. 76:101031. doi: 10.1016/j.destud.2021.101031

[ref55] Pauliuk S. Koslowski M. Madhu K. Schulte S. Kilchert S. (2022). Co-design of digital transformation and sustainable development strategies—what socio-metabolic and industrial ecology research can contribute. J. Clean. Prod. 343:130997. doi: 10.1016/j.jclepro.2022.130997

[ref56] Pivoto D. G. S. de Almeida L. F. F. da Rosa Righi R. Rodrigues J. J. P. C. Lugli A. B. Alberti A. M. (2021). Cyber-physical systems architectures for industrial internet of things applications in industry 4.0: A literature review. J. Manuf. Syst. 58, 176–192. doi: 10.1016/j.jmsy.2020.11.017

[ref57] Pyatt-Downes C. Kane G. (2019a). Using digital agile communities in industrial design. Des. J. 22(sup1), 2185–2188. doi: 10.1080/14606925.2019.1595008

[ref58] Pyatt-Downes C. Kane G. (2019b). Using digital agile communities in industrial desi.pdf. Available at: https://www.tandfonline.com/doi/pdf/10.1080/14606925.2019.1595008 (Accessed 5 March, 2022).

[ref59] Retolaza I. Ezpeleta I. Santos A. Diaz I. Martinez F. (2021). Design to cost; a framework for large industrial products. Procedia CIRP 100, 828–833. doi: 10.1016/j.procir.2021.05.036

[ref60] Rodríguez Ramírez E. R. (2014). Industrial design strategies for eliciting surprise. Des. Stud. 35, 273–297. doi: 10.1016/j.destud.2013.12.001

[ref61] Rosenzweig E. (2015). “Chapter 8: Iterating on the design,” in Successful User Experience: Strategies and Roadmaps. ed. Rosenzweig E. (Morgan Kaufmann), 155–176.

[ref62] Rossi F. Caloffi A. Colovic A. Russo M. (2022). New business models for public innovation intermediaries supporting emerging innovation systems: the case of the Internet of Things. Technol. Forecast. Soc. Chang. 175:121357. doi: 10.1016/j.techfore.2021.121357

[ref63] Schneorson D. Persov E. Bigger R. (2019). Designing your future—21st century skill-set for industrial designers. The case study of Israel Design Field. Des. J. 22(sup1), 243–259. doi: 10.1080/14606925.2019.1595862

[ref64] Selicati V. Intini F. Rospi G. Dassisti M. (2022). Addressing heterogeneity sources in manufacturing sustainability assessment using the system design view. CIRP J. Manuf. Sci. Technol. 37, 319–331. doi: 10.1016/j.cirpj.2022.02.009

[ref65] Shao Q. Chen L. Zhong R. Weng H. (2021). Marine economic growth, technological innovation, and industrial upgrading: A vector error correction model for China. Ocean Coast. Manag. 200:105481. doi: 10.1016/j.ocecoaman.2020.105481

[ref66] Shao X.-F. Liu W. Li Y. Chaudhry H. R. Yue X.-G. (2021). Multistage implementation framework for smart supply chain management under industry 4.0. Technol. Forecast. Soc. Chang. 162:120354. doi: 10.1016/j.techfore.2020.120354, PMID: 33041379 PMC7536173

[ref67] Sharma A. K. Bhandari R. Pinca-Bretotean C. Sharma C. Dhakad S. K. Mathur A. (2021). A study of trends and industrial prospects of industry 4.0. Mater. Today 47, 2364–2369. doi: 10.1016/j.matpr.2021.04.321

[ref68] Sinan Erzurumlu S. Erzurumlu Y. O. (2015). Sustainable mining development with community using design thinking and multi-criteria decision analysis. Resour. Policy 46, 6–14. doi: 10.1016/j.resourpol.2014.10.001

[ref69] Soni P. (2015). The Discovery of Grounded Theory (Glaser and Strauss, 1967).

[ref70] Sørensen B. M. (2008). ‘Behold, I am making all things new’: the entrepreneur as savior in the age of creativity. Scand. J. Manag. 24, 85–93. doi: 10.1016/j.scaman.2008.03.002

[ref71] Srinidhi N. N. Dilip Kumar S. M. Venugopal K. R. (2019). Network optimizations in the internet of things: a review. Eng. Sci. Technol. Int. 22, 1–21. doi: 10.1016/j.jestch.2018.09.003

[ref72] Stuedahl D. Lefkaditou A. Ellefsen G. S. Skåtun T. (2021). Design anthropological approaches in collaborative museum curation. Des. Stud. 75:101021. doi: 10.1016/j.destud.2021.101021

[ref73] Taneri B. Dogan F. (2021). How to learn to be creative in design: architecture students’ perceptions of design, design process, design learning, and their transformations throughout their education. Think. Skills Creat. 39:100781. doi: 10.1016/j.tsc.2020.100781

[ref74] Tiefenbacher K. F. (2019). “Chapter 8: New products require new thinking—ideas and examples,” in The Technology of Wafers and Waffles II. ed. Tiefenbacher K. F. (Academic Press), 131–220.

[ref75] Tseng M.-L. Bui T.-D. Lan S. Lim M. K. Mashud A. H. M. (2021). Smart product service system hierarchical model in banking industry under uncertainties. Int. J. Prod. Econ. 240:108244. doi: 10.1016/j.ijpe.2021.108244

[ref76] Urgo M. Terkaj W. Mondellini M. Colombo G. (2022). Design of serious games in engineering education: an application to the configuration and analysis of manufacturing systems. CIRP J. Manuf. Sci. Technol. 36, 172–184. doi: 10.1016/j.cirpj.2021.11.006

[ref77] Uskenbayeva R. K. Kuandykov A. Bolshibayeva A. Rakhmetulayeva S. B. (2020). An algorithm for creating an automated system based on platform of business process. Procedia Comput. Sci. 175, 253–260. doi: 10.1016/j.procs.2020.07.037

[ref78] Venturini F. (2022). Intelligent technologies and productivity spillovers: evidence from the fourth industrial revolution. J. Econ. Behav. Organ. 194, 220–243. doi: 10.1016/j.jebo.2021.12.018

[ref79] Wang L. Gao T. Zhou B. Tang H. Xiang F. (2022). Manufacturing service recommendation method toward industrial internet platform considering the cooperative relationship among enterprises. Expert Syst. Appl. 192:116391. doi: 10.1016/j.eswa.2021.116391

[ref80] Wang Y. Huang T. Wei G. Li H. Zhang H. (2022). Scalable name identifier lookup for Industrial Internet. Comput. Commun. 186, 102–109. doi: 10.1016/j.comcom.2022.01.017

[ref81] Wang B. Tao F. Fang X. Liu C. Liu Y. Freiheit T. (2021). Smart manufacturing and intelligent manufacturing: a comparative review. Engineering 7, 738–757. doi: 10.1016/j.eng.2020.07.017

[ref82] Warfield J. N. (1973). Binary matrices in system modeling. IEEE Trans. Syst. Man Cybern. SMC-3, 441–449. doi: 10.1109/TSMC.1973.4309270

[ref83] Wehrle M. Birkel H. von der Gracht H. A. Hartmann E. (2021). The impact of digitalization on the future of the PSM function managing purchasing and innovation in new product development—evidence from a Delphi study. J. Purch. Supply Manag.:100732. doi: 10.1016/j.pursup.2021.100732

[ref84] Xiao W. Cheng J. (2020). Perceptual design method for smart industrial robots based on virtual reality and synchronous quantitative physiological signals. Int. J. Distrib. Sens. Netw. 16:155014772091764. doi: 10.1177/1550147720917646

[ref85] Xu J. Hou Q. Niu C. Wang Y. Xie Y. (2018). Process optimization of the University-Industry-Research collaborative innovation from the perspective of knowledge management. Cogn. Syst. Res. 52, 995–1003. doi: 10.1016/j.cogsys.2018.09.020

[ref86] Xu R. Luo F. Chen G. Zhou F. Abdulahi E. W. (2021). Application of HFACS and grounded theory for identifying risk factors of air traffic controllers’ unsafe acts. Int. J. Ind. Ergon. 86:103228. doi: 10.1016/j.ergon.2021.103228

[ref87] Yoon Y. L. Yoon Y. Nam H. Choi J. (2021). Buyer-supplier matching in online B2B marketplace: an empirical study of small- and medium-sized enterprises (SMEs). Ind. Mark. Manag. 93, 90–100. doi: 10.1016/j.indmarman.2020.12.010

[ref88] Yuan C. Moon H. Wang S. Yu X. Kim K. H. (2021). Study on the influencing of B2B parasocial relationship on repeat purchase intention in the online purchasing environment: an empirical study of B2B E-commerce platform. Ind. Mark. Manag. 92, 101–110. doi: 10.1016/j.indmarman.2020.11.008

[ref95] Zhang L.-J. (2021). The area of Collaborative Innovation Study Industrial design service platform design research. Master’s Degree Thesis, Liaoning University of Science and Technology. https://kns.cnki.net/KCMS/detail/detail.aspx?dbname=CMFDTEMP&filename=1021169057.Nh

[ref89] Zhang X. Ming X. (2022). Implementation path and reference framework for Industrial Internet Platform (IIP) in product service system using industrial practice investigation method. Adv. Eng. Inform. 51:101481. doi: 10.1016/j.aei.2021.101481

[ref91] Zhang H. Zhang J. Song J. (2022). Analysis of the threshold effect of agricultural industrial agglomeration and industrial structure upgrading on sustainable agricultural development in China. J. Clean. Prod. 341:130818. doi: 10.1016/j.jclepro.2022.130818

[ref90] Zhang X. Ming X. Bao Y. Liao X. (2022). System construction for comprehensive industrial ecosystem oriented networked collaborative manufacturing platform (NCMP) based on three chains. Adv. Eng. Inform. 52:101538. doi: 10.1016/j.aei.2022.101538

[ref01] Zheng J. Shao X. Liu W. Kong J. Zuo G. (2021). The impact of the pilot program on industrial structure upgrading in low-carbon cities. J. Clean. Prod. 290:125868. doi: 10.1016/j.jclepro.2021.125868

[ref92] Zhu W. Zhu Y. Lin H. Yu Y. (2021). Technology progress bias, industrial structure adjustment, and regional industrial economic growth motivation—research on regional industrial transformation and upgrading based on the effect of learning by doing. Technol. Forecast. Soc. Chang. 170:120928. doi: 10.1016/j.techfore.2021.120928

[ref93] Zivkovic Z. Nikolic S. T. Doroslovacki R. Lalic B. Stankovic J. Zivkovic T. (2015). Fostering creativity by a specially designed Doris tool. Think. Skills Creat. 17, 132–148. doi: 10.1016/j.tsc.2015.06.004

[ref94] Zuljevic M. Huybrechts L. (2021). Historicising design space: uses of the past in participatory prefiguring of spatial development. Des. Stud. 73:100993. doi: 10.1016/j.destud.2021.100993

